# The Role of EFL/ESL Teachers’ Psychological Empowerment and Optimism on Their Job Commitment

**DOI:** 10.3389/fpsyg.2022.941361

**Published:** 2022-06-24

**Authors:** Xiaoqin Xiong

**Affiliations:** School of Foreign Studies, China University of Petroleum, Qingdao, China

**Keywords:** EFL/ESL teachers, psychological empowerment, optimism, commitment, L2 education

## Abstract

Research has approved that teaching is a complex profession involving many cognitive, social, cultural, and psycho-emotional factors. To perform efficiently, teachers must be psycho-emotionally powerful and ready to cope with the existing challenges and complications of teaching a second/foreign language. This demands attempts to be made to psychologically empower the teachers to form positive outlooks about their profession and practices. Despite the criticality of psychological empowerment (PE), few studies in L2 contexts have dealt with it. Against this gap, the present article aimed to theoretically analyze the interaction among teachers’ PE, optimism, and commitment. In so doing, the definitions, models, components, typologies, and empirical studies related to these constructs were presented. Finally, practical implications of this line of research for EFL/ESL teachers, teacher trainers, and researchers are provided to raise their awareness of psycho-emotional factors involved L2 education.

## Introduction

Teaching has long been dismissed as a demanding profession because of involving numerous inner and outer factors (Benevene et al., 2020). Now, the cognitive perspective that had dominated education has been complemented by trends considering various psycho-emotional and social variables (Agudo, 2018; Mercer, 2020). Owing to their considerable impacts, inner states and psychological factors can influence every single aspect of one’s job including performance, satisfaction, behavior, and classroom practices (Sikma, 2021). Yet, in the context of second/foreign language education, the story gets more complicated in the sense that working in a language other than one’s native language places many emotional tensions and pressures on the teacher to generate positive academic outcomes (King and Ng, 2018; Wang and Derakhshan, 2021). Such invisible emotional challenges are contended to prevent the teachers to play their crucial role in academia and, ultimately, the quality of education collapses (Asrar-ul-Haq et al., 2017). To compensate for the inadequacies in supporting teachers and their services, in the past decades effective attempts have been made to understand and consider EFL/ESL teachers’ psychological and emotional health (Khany and Tazik, 2015). An insightful way is psychologically empowering the teachers by giving them an opportunity to autonomously make decisions, practice, behave, and fulfill duties in the classroom (Ford and Fottler, 1995; Kõiv et al., 2019). The concept of psychological empowerment (PE) is rooted in business, yet explained and used in education with the seminal work of Bandura (1997) on self-efficacy theory. It refers to a teacher’s self-belief in the work role and the perception of his/her autonomy, self-competence, agency, and meaning in the work he/she does (Simonet et al., 2015; Ahmed and Malik, 2019). PE has gained a considerable amount of scholarly attention in the past years revealing that the variable plays a critical role in teaching and teacher quality, creativity, performance, adaptability, satisfaction, motivation, wellbeing, and so forth. Concerning students, teachers’ PE has been identified to contribute to students’ success and innovative behaviors in the class (Çelik and Servet, 2020).

Another factor that is in a close relationship with PE is optimism (Valeh et al., 2021). By definition, optimism is one’s positive expectation about the future in spite of the existing difficulties and setbacks (Carver and Scheier, 2002). It is a personal tendency to consider that one will experience good occasions in life and survive bad outcomes (Dong and Xu, 2022). In teaching, optimism means teachers’ positive view about his/her ability to generate academic success among students despite challenges (Pathak and Lata, 2018). Optimistic teachers have been approved to enjoy high levels of resilience, efficacy, enthusiasm, and work engagement (Lu, 2021; Dong and Xu, 2022). When a teacher is psychologically empowered, by schools and administrators, his/her optimism level increases, too, and many academic outcomes emerge due to teacher’s mental strengths and positive views of the future.

A further area that teachers’ PE and optimism can affect is their job commitment or the degree to which teachers are happy about their work and strive to demonstrate improved job execution (Altun, 2017). It is a sense of belongingness and loyalty to one’s profession that determines his/her performance, attendance, motivation, satisfaction, persistence, and disposition toward success (Skaalvik and Skaalvik, 2011). Commitment is a mental connection between one’s job and his/her faiths, beliefs, goals, and practices (Lu, 2021). Despite the significance of these three variables (i.e., PE, optimism, and commitment) in L2 education, limited studies have been done on their interaction. Against this backdrop, the present article aimed to provide a theoretical review of the definitions, dimensions, features, and studies related to EFL/ESL teachers’ PE, optimism, and job commitment.

## Background

### The Concept of Psychological Empowerment

The concept of PE found its way from business to education in 1990s (Çelik and Servet, 2020). It has become the foci of educational practitioners and institutions to improve their human resources at work (Degago, 2014). While the term may have different conceptualizations across contexts (Zimmerman, 1990), it has been widely regarded as a sense of self-belief, competence, and autonomy that a person has in the workplace (Ahmed and Malik, 2019). It is both a process and an outcome that can occur at individual, organizational, and community levels (Li, 2016). PE is an essential factor in determining many work-related outcomes that increases the level of motivation, agency, and self-determination in doing tasks and duties at work (Chan et al., 2015; Kõiv et al., 2019). PE is related to the motivational aspects of work or employee’s intrinsic motivation to work or behave with agency, confidence, and authority (Singh and Kaur, 2019). To put it simply, PE is an inner state of a person in which he/she is fully aware of his/her duties, strengths, and skills to grow personally and professionally. It determines the functionality, commitment, performance, and satisfaction of the individual at workplace if it is considered and supported by academic staff and administrators (Khany and Tazik, 2015).

### The Dimensions of Psychological Empowerment

Psychological empowerment has been considered as a multi-dimensional construct in education due to its multi-layered nature (Singh and Kaur, 2019). In this regard, Short and Rinehart (1992) argued that the construct of PE has six dimensions including; professional development, status, impact, autonomy, decision-making, and self-efficacy. Likewise, Spreitzer (1995) proposed three other dimensions to PE, namely competence, meaning, and self-determination. Impact concerns teachers’ perceived power to influence the decision-making and practices of the school, while meaning pertains to the joy that teachers experience when their beliefs and values fit together. Moreover, competence refers to a teacher’s belief in his/her abilities to accomplish a task or duty properly. Lastly, self-determination concerns teachers’ control over school’s instructional plans, decisions, and activities (Çelik and Servet, 2020).

### Models of Psychological Empowerment

Despite the growing interest in researching the concept of PE in education, limited models have been proposed for it. Yet, the most widely used and cited model of PE is that of Zimmerman (1995) which posits that PE is made up of three components; *intrapersonal*, *interactional*, and *behavioral* empowerment. The first component of this model concerns how people think about themselves and their ability to affect others along with the socio-political systems (Petrovčič and Petrič, 2014). It comprises three sub-dimensions of *control*, *self-efficacy* and *perceived competence* (Leung, 2009). Control is associated to one’s beliefs about his/her ability to affect the environment, while self-efficacy concerns an individual’s self-assessment of his/her abilities to accomplish specific tasks. Finally, perceived competence refers to one’s perceptions of his/her capacity to carry out a job/task efficiently (Zimmerman, 2000; Hur, 2006).

The second component, interactional, refers to an individual’s intellectual understanding of the immediate social context and the knowledge and resources needed to make changes there (Zimmerman, 1995). It also capitalizes on the awareness of the existing choices to reach goals and understand the norms and values of a specific setting. This component is believed to develop one’s decision-making and problem-solving skills that lead to engagement in the context/work (Zimmerman, 2000). Finally, the behavioral component of PE pertains to the actions that may affect outcomes after being empowered (Zimmerman, 1995). This is accomplished by partaking in activities and community organizations including different political, self-help, and religious groups or organizations that help deal with problems (Zimmerman et al., 1992).

**FIGURE 1:**
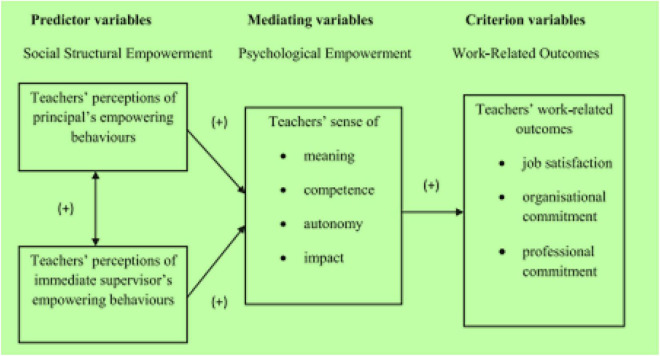
A Theoretical model of teacher empowerment (Lee and Nie, 2014, p. 72).

Drawing on Zimmerman’s (1995) model, Lee and Nie (2014) made a seminal attempt to integrate the social-structural and psychological views of empowerment into a solid theoretical framework for teacher PE in Singapore. The model depicted the predictive relationship between the social-structural and psychological perspectives of PE and work-related outcomes (Figure 1).

Although these two models have provided valuable insights into the construct of teacher PE, their application in EFL/ESL contexts has not been scientifically tested. Moreover, how each of the components or dimensions of these models can be practiced by teachers to expand their PE needs more research in the future.

### Optimism: Definitions and Dimensions

The concept of optimism is a psycho-emotional construct that took its roots in Bandura’s social intellectual hypothesis, but boomed in positive psychology as a novel trend in educational psychology (Dong and Xu, 2022). It is regarded as a propensity to expect positive outcomes (Srivastava and Angelo, 2009). It has also been seen as an intrinsic attribute that highlights one’s prediction and expectation of positive events and outcomes in the future despite difficulties and challenges (Carver and Scheier, 2002). It is generally conceptualized as the mirror image of pessimism in education and psychology (Srivastava and Angelo, 2009). According to Pathak and Lata (2018), optimist people are characterized by high degrees of resilience, self-esteem, extraversion, motivation and low levels of stress, anxiety, and hopelessness.

**FIGURE 2:**
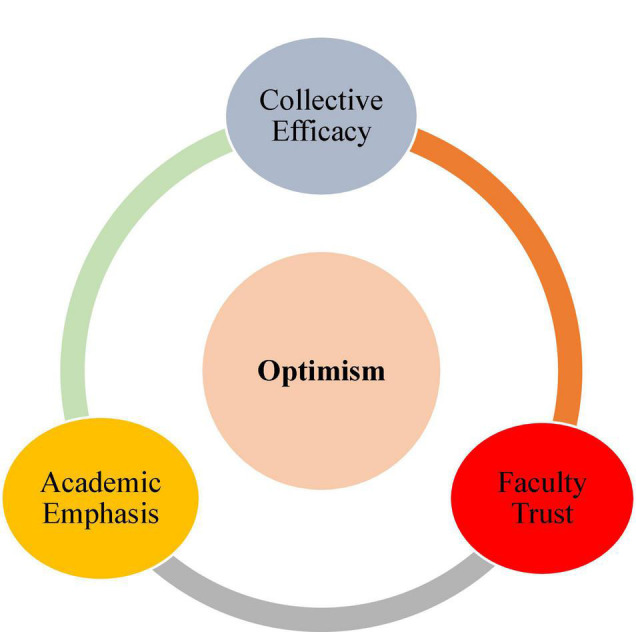
The dimensions of optimism (Hoy et al., 2006).

To paint a more vivid picture of optimism, three dimensions or layers have been offered for the construct of teacher optimism. They comprise *academic emphasis, faculty trust*, and *collective efficacy* as depicted in Figure 2. The first dimension has to do with teachers’ enacted behavior stimulated by their beliefs in producing learning and success in learners through an optimistic classroom environment. Faculty trust, as the second dimension, pertains to teachers’ assurance in engaging students and parents in the learning process. This immersion contributes to setting high standards for learning which are certified and preferred by students as well as parents. As the final dimension, collective efficacy concerns teachers’ belief in their capability to implement teaching efficiently and professionally to produce academic achievement among students (Hoy et al., 2006; Hoy and Miskel, 2013). It is noteworthy that these three dimensions are not mutually exclusive but in interaction with one another to get shaped (Dong and Xu, 2022).

### Types of Optimism

In the available literature on optimism in educational psychology, different typologies have been offered to the construct of optimism. On the basis of endurance, optimism has been divided into *state* optimism and *trait* optimism. State or situational optimism refers to the expectation of desirable outcomes in specific situations. In contrast, trait optimism is a more steady expectation of good outcomes across different contexts and times (Tusaie and Patterson, 2006; Carver and Scheier, 2014; Wang et al., 2018). Other types of optimism include dispositional or global, attributional, realistic, unrealistic, comparative, and strategic. Dispositional or global optimism (also known as big optimism) is a stable and global expectation that more good things than bad will occur in the future (Scheier and Carver, 1985; Peterson, 2000; Carver and Scheier, 2014). Attributional optimism has to do with the style of reasoning about the cause of events (Buchanan and Seligman, 1995; Gordeeva et al., 2020). Accordingly, optimistic people usually consider good events to be permanent, pervasive, and internal to self. On the contrary, bad happenings are temporary, non-pervasive, and attributable to external causes.

Realistic optimism is the propensity to uphold a positive outlook in the boundaries of the physical and social world in measuring an event (Schneider, 2001). Unrealistic optimism, in contrast, refers to an illusion that happens when a person improperly predicts more positive outcomes to occur to him/her than others (Shepperd et al., 2013). Additionally, comparative optimism pertains to predicting and expecting more good outcomes occurring to the self than for others (Radcliffe and Klein, 2002). Last but not least, strategic optimism refers to the expectation of desirable outcomes and events without fearing or caring too much about the likelihood of adverse events (Ruthig et al., 2007; Bunjak and Èerne, 2018).

### The Concept of Teacher Commitment in Education

The concept of commitment is described as one’s sense of belonging and loyalty to the organization and profession in which he/she is working (Skaalvik and Skaalvik, 2011). It is composed of three factors of identification, involvement, and loyalty to a job (Pourtousi and Ghanizadeh, 2020). As pinpointed by Meyer and Allen (1991), commitment to an organization can also be conceptualized as affective commitment, continuance commitment, and normative commitment. The first type of commitment to a job intends to create a sense of comfort or having affection for the job. The second one refers to the degree to which an individual feels the need to stay at an organization. It is also characterized as the fear of losing a job. Finally, normative commitment mirrors a sense of obligation to stay loyal to a job (Meyer and Allen, 1991). In other words, commitment to education concerns teachers’ desire to work and go about creating great and successful instruction (Day, 2004). As the wellspring of inspiration and the core of quality schooling, commitment is a fundamental element for optimum instruction that includes commitment to the school, students, occupation maintenance, proficient knowledge base, and teaching career (Crosswell and Elliott, 2004). A growing bulk of research has indicated that teacher commitment is affected by many internal and external factors in order to reduce teacher turnover, make curricular developments, approve change in a discipline, correspond to program advancement, sustain achievement, and increase the profundity of students’ development (Ingersoll and May, 2012; Robinson and Edwards, 2012; Mee and Haverback, 2014; Sorensen and McKim, 2014; McKim and Velez, 2016). This construct has been identified and approved to be one of the most essential variables for the future success of education at all levels (Pourtousi and Ghanizadeh, 2020). To make commitment possible in educational contexts, administrators and principals need to establish a positive and caring climate in which teachers feel safe and appreciated so that they can stay loyal to the school and the job.

### The Characteristics of a Committed Teacher

There are several features characterizing a teacher who has a commitment to his/her profession. A committed teacher is passionate and zealous about teaching and students’ learning (Mustafa et al., 2020). Such teachers try to inspire their pupils to be more willing to achieve. Moreover, teachers with commitment provide their students with imaginative instructional techniques that can generate higher degree of success and eagerness to learn among students (Fink, 2003). Additionally, committed teachers are powerful in producing energy and engagement in the classroom by motivating students to be involved in school exercises (Lu, 2021). Another feature of a committed teacher is being receptive to others’ ideas and beliefs that help him/her execute the task of teaching more efficiently (Huang et al., 2016; Altun, 2017). The ability to establish an operational learning environment for students to learn and academically improve is another feature of a committed teacher (Peterson and Skiba, 2000). Likewise, a teacher with commitment has the enthusiasm to take extra tasks and duties in his/her obligations to meet long-term and short-term objectives of the school (Sarikaya and Erdogan, 2016). Moreover, committed teachers have strong mental connections with their job, work with affection, are dedicated to the faculty, constantly endeavor to improve, seek greatness in executing their duty, and are adroit in interpersonal communication skills (Dannetta, 2002; Smith, 2010; Lu, 2021; Xie and Derakhshan, 2021).

### The Measurement of Psychological Empowerment, Optimism, and Commitment

In order to measure the psycho-emotional constructs of PE, optimism, and commitment, the extant literature has mainly used questionnaires and qualitative research instruments are scant, if any. With regard to PE, the most popular scale was designed and validated by Spreitzer (1995) including 12 items under four sub-categories of meaning, competence, self-determination, and impact that assess the perceptions of PE. It follows a 5-point Likert scale enjoying high degrees of reliability (0.80) and validity as approved by more than 50 studies (Amundsen and Martinsen, 2015; Gong et al., 2020). More recently, Van Dop et al. (2016) developed a new questionnaire for measuring PE using Zimmerman’s (1995) theory of PE. It is comprised of 28 items presented in a 5-point Likert scale and divided into three dimensions of intrapersonal, interactional, and behavioral dimensions.

In a similar vein, the measurement of optimism has been largely done by scales including the Expanded Attributional Style Questionnaire developed by Peterson and Villanova (1988), the Life Orientation Test (Scheier and Carver, 1985), and the Vision about the Future scale (Ginevra et al., 2016). Likewise, Hoy et al. (2008) developed a comprehensive scale including 40 items measuring academic optimism on the basis of a 5-point Likert scale. Nevertheless, some scholars in the past decade began measuring optimism qualitatively using interviews (e.g., Nota et al., 2015) yet this area is still limited to self-reported scales.

Similarly, available studies on teacher commitment have benefited from questionnaires to assess the level of commitment among their participants. The most common scales include that of Thien et al. (2014) who validated a scale for teacher commitment in Malaysia. Their scale had 13 items spread along four dimensions (i.e., Commitment to School, Commitment to Students, Commitment to Teaching, and Commitment to Profession) following a 6-point Likert scale. Another scale to assess teacher commitment is the Vocational Exploration and Commitment Scale designed by Blustein et al. (1989) that includes 19 items presented in a 7-point Likert scale. Likewise, teacher commitment has been measured *via* the Work Commitment Index proposed by Blau et al. (1993). This questionnaire encompasses 17 items on a 6-point Likert scale assessing the personal, emotional, and motivational commitment of teachers. In addition to these quantitative tools, prompts, open-ended questions, and scenarios have also been used to measure teacher commitment (Chesnut, 2017). Still, the use of qualitative research instruments to measure these three constructs is its infancy calling for more empirical studies across the world.

### Empirical Studies

After the crystallization of the constructs of PE, optimism, and commitment together with the development of research instruments to gauge them, many empirical studies have been carried out to unpack their relationship with other psych-emotional variables. As for teacher PE, research indicates that it is correlated with psychological capital, job involvement, satisfaction, retention, work performance, motivation, creativity, leadership, autonomy, self-determination, wellbeing, self-efficacy, and work attachment (Degago, 2014; Khany and Tazik, 2015; Ahmed and Malik, 2019; Kõiv et al., 2019; Singh and Kaur, 2019; Çelik and Servet, 2020; Ma et al., 2021). Moreover, in Iran, Rezaei et al. (2015) ran a correlational study with 200 primary school teachers and identified that PE has a significant and positive relationship with psychological capital and its dimensions. Likewise, in their recent study, Tsang et al. (2022) distributed a questionnaire among 322 primary and secondary school teachers in China and found that PE is negatively correlated with teacher burnout. Moreover, they identified that two dimensions of PE (i.e., meaning, competence) mediated the association between structural empowerment and teacher burnout. A problem with the current studies is that they have mostly utilized one-shot, correlational research designs without exploring the depth of the relationship between PE and teacher-related variables.

Another area that teacher PE can influence or be associated with is teacher optimism or having a positive outlook of the future. Although it is axiomatic that when a person is psychologically empowered he/she is more likely to have a positive view of the future than someone who is psychologically weak at workplace, few studies (if any) have strived to test their relationship. Yet, the construct of optimism has been approved to increase teachers’ resilience, efficacy, enthusiasm, and work engagement (Lu, 2021; Dong and Xu, 2022). Both teacher PE and optimism have also been found to affect several teachers’ work-related outcomes like job satisfaction, and commitment (Lee and Nie, 2014). However, most of the existing body of research has gleaned the pertinent data *via* qualitative instruments in a short period of time. This necessitates the conduction of qualitative and mixed-methods studies.

Additionally, research shows that teacher commitment has a close relationship with self-regulation, academic success, motivation, and self-efficacy (Royaei and Ghanizadeh, 2016; Chesnut, 2017; Pourtousi and Ghanizadeh, 2020). Likewise, teacher commitment to teaching has been claimed to counterbalance the desire to quit the job (Klassen and Chiu, 2011). Despite these correlational studies signifying the association among teacher PE, optimism, commitment many psycho-emotional variables, the way these three factors operationalize and work in EFL/ESL contexts have been ignored, to date. In other words, how and in what ways a psychologically empowered teacher can elevate his/her optimism and commitment to teaching is not still scientifically clear. To cast some light on this gap, the present article was a bid to theoretically analyze these constructs and their interaction in an effort to add fresh insights to the intersection of teacher psychology and teacher education in EFL/ESL milieus.

### Concluding Remarks

In this theoretical review, it was argued that EFL/ESL teachers’ PE and optimism have a close relationship with commitment to work. The belief is that psychologically empowered L2 teachers are more resourceful in dealing with many challenges of the profession. Hence, they are resilient and optimistic about the coming events of their job. As they are mentally powerful, they see the positive sides of their job and feel a sense of belonging and loyalty to teaching. Based on these, it is argued that this theoretical review is of help to L2 teachers who realize the impact and role of psych-emotional factors in their occupation and instruction. They can work on activities that boost their mental strengths that generate many other positive outcomes. Teacher trainers can also use the ideas pinpointed in this review to design and offer professional development programs to EFL/ESL teachers in order to psychologically and emotionally empower them before or during their teaching career. In such courses, trainers can present EFL/ESL teachers various techniques and activities through which the teachers can maintain and develop their level of PE, optimism, and commitment. Moreover, L2 scholars can use this study as a starting point to further explore the interaction of the three variables covered in this study using qualitative instruments such as interviews, diaries, audio journals, portfolios, and observations. Moreover, cross-cultural studies can be conducted to see the role of cultural factors (e.g., individualism, collectivism, adaptability, socialization, cultural identity, ethnicity, etc.) in determining these constructs. Future research can also compensate for the current limitations in this area, namely self-reported data, self-flattery of participants, small sample size, and limited generalizability of associational studies. Finally, further studies are recommended to develop models for the implementation of teacher PE, optimism, and commitment specialized for L2 education which involves many complications.

## Author Contributions

The author confirms being the sole contributor of this work and has approved it for publication.

## Conflict of Interest

The author declares that the research was conducted in the absence of any commercial or financial relationships that could be construed as a potential conflict of interest.

## Publisher’s Note

All claims expressed in this article are solely those of the authors and do not necessarily represent those of their affiliated organizations, or those of the publisher, the editors and the reviewers. Any product that may be evaluated in this article, or claim that may be made by its manufacturer, is not guaranteed or endorsed by the publisher.

## References

[B1] Agudo J. D. M. (2018). *Emotions In Second Language Teaching: Theory, Research And Teacher Education.* Cham: Springer.

[B2] Ahmed N. Malik B. (2019). Impact of psychological empowerment on job performance of teachers: mediating role of psychological wellbeing. *Rev. Econ. Dev. Stud.* 5 451–460. 10.26710/reads.v5i3.693

[B3] Altun M. (2017). The effects of teacher commitment on student achievement: a case study in Iraq. *Int. J. Acad. Res. Bus. Soc. Sci.* 7 417–426. 10.6007/IJARBSS/v7-i11/3475

[B4] Amundsen S. Martinsen Ø. L. (2015). Linking empowering leadership to job satisfaction, work effort, and creativity: the role of self-leadership and psychological empowerment. *J. Leadersh. Organ. Stud.* 22 304–323. 10.1177/1548051814565819

[B5] Asrar-ul-Haq M. Anwar S. Hassan M. (2017). Impact of emotional intelligence on teacher’s performance in higher education institutions of Pakistan. *Futur. Bus. J.* 3 87–97. 10.1016/j.fbj.2017.05.003

[B6] Bandura A. (1997). *Self-Efficacy: The Exercise Of Control.* New York: Freeman.

[B7] Benevene P. De Stasio S. Fiorilli C. (2020). Wellbeing of school teachers in their work environment. *Front. Psychol.* 11:1239. 10.3389/fpsyg.2020.01239 32903655 PMC7438736

[B8] Blau G. Paul A. St. John N. (1993). On developing a general index of work commitment. *J. Vocat. Behav.* 42:298e314. 10.1006/jvbe.1993.1021

[B9] Blustein D. L. Ellis M. V. Devenis L. E. (1989). The development and validation of a two-dimensional model of the commitment to career choices process. *J. Vocat. Behav.* 35 342–378.

[B10] Buchanan G. M. Seligman M. E. P. (1995). *Explanatory Style.* Hillsdale, NJ: Erlbaum.

[B11] Bunjak A. Èerne M. (2018). Mindfulness–The missing link in the relationship between leader–follower strategic optimism (mis) match and work engagement. *Front. Psychol.* 9:2444. 10.3389/fpsyg.2018.02444 30564180 PMC6288291

[B12] Carver C. S. Scheier M. F. (2002). “Optimism,” in *Handbook Of Positive Psychology*, eds Snyder C. R. Lopez S. J. (New York: Oxford University Press), 231–243.

[B13] Carver C. S. Scheier M. F. (2014). Dispositional optimism. *Trends Cogn. Sci.* 18 293–299. 24630971 10.1016/j.tics.2014.02.003 PMC4061570

[B14] Çelik O. T. Servet A. T. I. K. (2020). Preparing teachers to change: the effect of psychological empowerment on being ready for individual change. *Cukurova University Fac. Educ. J.* 49 73–97.

[B15] Chan Y. H. Scott Nadler S. Hargis M. B. (2015). Attitudinal and behavioral outcomes ofemployees’ psychological empowerment: a structural equation modeling approach. *J. Organ. Cult. Commun. Confl.* 19 24–41.

[B16] Chesnut S. R. (2017). On the measurement of pre-service teacher commitment: examining the relationship between four operational definitions and self-efficacy beliefs. *Teach. Teach. Educ.* 68 170–180. 10.1016/j.tate.2017.09.003

[B17] Crosswell L. Elliott R. (2004). “Committed teachers, passionate teachers: The dimension of passion associated with teacher commitment and engagement,” in *AARE Conference 2004*, ed. Ruth J. (Melbourne: Australian Association for Research in Education), 1–12.

[B18] Dannetta V. (2002). What factors influence a teacher’s commitment to student learning? *Leadersh. Policy Sch.* 1 144–171.

[B19] Day C. (2004). The passion of successful leadership. *Sch. Leadersh. Manag.* 24 425–437. 10.1080/13632430410001316525

[B20] Degago E. (2014). A study on impact of psychological empowerment on employee performance in small and medium scale enterprise sectors. *Eur. J. Bus. Manag.* 6 60–72.

[B21] Dong Y. Xu J. (2022). The role of EFL teachers’ optimism and commitment in their work engagement: a theoretical review. *Front. Psychol.* 12:830402. 10.3389/fpsyg.2021.830402 35185712 PMC8851313

[B22] Fink L. D. (2003). *Creating Significant Learning Experiences.* San Francisco, CA: Jossey Bass.

[B23] Ford R. C. Fottler M. D. (1995). Empowerment: a matter of degree. *Acad. Manag. Exec.* 9 21–29.

[B24] Ginevra M. C. Pallini S. Vecchio G. M. Nota L. Soresi S. (2016). Future orientation and attitudes mediate career adaptability and decidedness. *J. Vocat. Behav.* 95 102–110.

[B25] Gong Y. Wu Y. Huang P. Yan X. Luo Z. (2020). Psychological empowerment and work engagement as mediating roles between trait emotional intelligence and job satisfaction. *Front. Psychol.* 11:232. 10.3389/fpsyg.2020.00232 32210866 PMC7067919

[B26] Gordeeva T. Sheldon K. Sychev O. (2020). Linking academic performance to optimistic attributional style: attributions following positive events matter most. *Eur. J. Psychol. Educ.* 35 21–48. 10.1007/s10212-019-00414-y

[B27] Hoy A. W. Hoy W. K. Kurz N. M. (2008). Teacher’s academic optimism: the development and test of a new construct. *Teach. Teach. Educ.* 24 821–835.

[B28] Hoy W. K. Miskel C. G. (2013). *Educational Administration: Theory, Research And Practice.* New York, NY: McGraw Hill.

[B29] Hoy W. K. Tarter C. J. Hoy A. W. (2006). Academic optimism of schools: a force for student achievement. *Am. Educ. Res. J.* 43 425–446.

[B30] Huang X. Lee J. C. K. Zhang Z. Wang J. (2016). “Teacher commitment in northwest China,” in *Educational Development In Western China*, eds Lee J. C. K. Yu Z. Huang X. Law E. H. F. (Rotterdam: SensePublishers), 261–275. 10.3389/fpsyg.2021.729504

[B31] Hur M. H. (2006). Empowerment in terms of theoretical perspectives: exploring a typology of the process and components across disciplines. *J. Community Psychol.* 34 523–540. 10.1002/jcop.20113

[B32] Ingersoll R. M. May H. (2012). The magnitude, destinations, and determinants of mathematics and science teacher turnover. *Educ. Eval. Policy Anal.* 34 435–464.

[B33] Khany R. Tazik K. (2015). On the relationship between psychological empowerment, trust, and Iranian EFL teachers’ job satisfaction: the case of secondary school teachers. *J. Career Assess.* 24 112–129. 10.1177/1069072714565362

[B34] King J. Ng K.-Y. S. (2018). “Teacher emotions and the emotional labour of second language teaching,” in *Language Teacher Psychology*, eds Mercer S. Kostoulas A. (Bristol: Multilingual Matters), 141–157.

[B35] Klassen R. M. Chiu M. M. (2011). The occupational commitment and intention toquit of practicing and pre-service teachers: influence of self-efficacy, job stress, and teaching context. *Contemp. Educ. Psychol.* 36 114–129. 10.1016/j.cedpsych.2011.01.002

[B36] Kõiv K. Liik K. Heidmets M. (2019). School leadership, teacher’s psychological empowerment and work-related outcomes. *Int. J. Educ. Manag.* 33 1501–1514. 10.1108/IJEM-08-2018-0232

[B37] Lee A. N. Nie Y. (2014). Understanding teacher empowerment: teachers’ perceptions of principal’s and immediate supervisor’s empowering behaviors, psychological empowerment, and work-related outcomes. *Teach. Teach. Educ.* 41 67–79. 10.1016/j.tate.2014.03.006

[B38] Leung L. (2009). User-generated content on the Internet: an examination of gratifications, civic engagement and psychological empowerment. *New Media Soc.* 11 1327–1347.

[B39] Li Z. (2016). Psychological empowerment on social media: who are the empowered users? *Public Relat. Rev.* 42 49–59.

[B40] Lu D. (2021). EFL teachers’ optimism and commitment and their contribution to students’ academic success. *Front. Psychol.* 12:752759. 10.3389/fpsyg.2021.752759 34733218 PMC8558303

[B41] Ma L. Zhou F. Liu H. (2021). Relationship between psychological empowerment and the retention intention of kindergarten teachers: a chain intermediary effect analysis. *Front. Psychol.* 12:248. 10.3389/fpsyg.2021.601992 33679521 PMC7928276

[B42] McKim A. J. Velez J. J. (2016). An evaluation of the self-efficacy theory in agricultural education. *J. Agric. Educ.* 57 73–90.

[B43] Mee M. Haverback H. R. (2014). Commitment, preparation, and early career frustrations: examining future attrition of middle school teachers. *Am. Second. Educ.* 42 39–51.

[B44] Mercer S. (2020). The wellbeing of language teachers in the private sector: an ecological perspective. *Lang. Teach. Res.* 1 1–24.

[B45] Meyer J. P. Allen N. J. (1991). A three-component conceptualization of organizational commitment. *Hum. Resour. Manag. Rev.* 1 61–89. 10.1016/1053-4822

[B46] Mustafa M. Z. B. Nordin M. B. Razzaq A. R. B. A. bin Ibrahim B. (2020). Organizational Commitment of Vocational College Teachers in Malaysia. *PalArch’s J. Archaeol. Egypt/Egyptol.* 17 5023–5029.

[B47] Nota L. Santilli S. Soresi S. (2015). “Qualitative approaches to career assessment with people with disability,” in *Career Assessment*, eds McMahon M. Watson M. (Rotterdam: Brill Sense), 221–229. 10.1007/978-94-6300-034-5_25

[B48] Pathak R. Lata S. (2018). Optimism in relation to resilience and perceived stress. *J. Psychosoc. Res.* 13 359–367. 10.32381/JPR.2018.13.02.10

[B49] Peterson C. (2000). The future of optimism. *Am. Psychol.* 55 44–55. 10.1037/0003-066X.55.1.44 11392864

[B50] Peterson C. Villanova P. (1988). An expanded attributional style questionnaire. *J. Abnorm. Psychol.* 97 87–89. 10.1037/0021-843X.97.1.87 3351118

[B51] Peterson R. L. Skiba R. (2000). Creating school climates that prevent school violence. *Prev. Sch. Fail.* 44 122–129. 10.1080/10459880009599794

[B52] Petrovčič A. Petrič G. (2014). Differences in intrapersonal and interactional empowerment between lurkers and posters in health-related online support communities. *Comput. Hum. Behav.* 34 39–48. 10.1016/j.chb.2014.01.008

[B53] Pourtousi Z. Ghanizadeh A. (2020). Teachers’ motivation and its association with job commitment and work engagement. *Psychol. Stud.* 65 455–466. 10.3389/fpsyg.2022.850204 35558709 PMC9087840

[B54] Radcliffe N. M. Klein W. M. (2002). Dispositional, unrealistic, and comparative optimism: differential relations with the knowledge and processing of risk information and beliefs about personal risk. *Pers. Soc. Psychol. Bull.* 28 836–846.

[B55] Rezaei M. Hoveida R. Samavatian H. (2015). Concept of psychological empowerment and its relationship with psychological capital among teachers. *New Educ. Approaches* 10 67–82. 10.1186/s12913-016-1423-5 27409075 PMC4943498

[B56] Robinson J. S. Edwards M. C. (2012). Assessing the teacher self-efficacy of agriculture instructors and their early career employment status: a comparison of certification types. *J. Agric. Educ.* 53 150–161. 10.5032/jae.2012.01150

[B57] Royaei N. Ghanizadeh A. (2016). The interface between motivational and emotional facets of organizational commitment among instructors at higher education. *RIMCIS* 5 228–252. 10.17583/rimcis.2016.2139

[B58] Ruthig J. C. Haynes T. L. Perry R. P. Chipperfield J. G. (2007). Academic optimistic bias: implications for college student performance and wellbeing. *Soc. Psychol. Educ.* 10 115–137. 10.1007/s11218-006-9002-y

[B59] Sarikaya N. Erdogan Ç (2016). Relationship between the instructional leadership behaviors of high school principals and teachers’ organizational commitment. *J. Educ. Pract.* 7 72–82.

[B60] Scheier M. F. Carver C. S. (1985). Optimism, coping, and health: assessment and implications of generalized outcome expectancies. *Health Psychol.* 4 219247. 10.1037//0278-6133.4.3.219 4029106

[B61] Schneider S. L. (2001). In search of realistic optimism: meaning, knowledge, and warm fuzziness. *Am. Psychol.* 56 250–263. 10.1037//0003-066x.56.3.250 11315251

[B62] Shepperd J. A. Klein W. M. Waters E. A. Weinstein N. D. (2013). Taking stock of unrealistic optimism. *Perspect. Psychol. Sci.* 8 395–411. 10.1177/1745691613485247 26045714 PMC4451212

[B63] Short P. M. Rinehart J. S. (1992). School participant empowerment scale: assessment of level of empowerment within the school environment. *Educ. Psychol. Meas.* 52 951–960. 10.1177/0013164492052004018

[B64] Sikma L. (2021). “Building resilience: Using BRiTE with beginning teachers in the United States,” in *Cultivating Teacher Resilience*, ed. Mansfield C. F. (Singapore: Springer), 85–101. 10.1007/978-981-15-5963-1_6

[B65] Simonet D. V. Narayan A. Nelson C. A. (2015). A social-cognitive moderated mediated model of psychological safety and empowerment. *J. Psychol.* 149 818–845. 10.1080/00223980.2014.981496 25511012

[B66] Singh K. Kaur S. (2019). Psychological empowerment of teachers: development and validation of multi-dimensional scale. *Int. J. Recent Technol. Eng.* 7 340–343.

[B67] Skaalvik E. M. Skaalvik S. (2011). Teacher job satisfaction and motivation to leave the teaching profession: relations with school context, feeling of belonging, and emotional exhaustion. *Teach. Teach. Educ.* 27 1029–1038. 10.1016/j.tate.2011.04.001

[B68] Smith E. (2010). Underachievement, failing youth and moral panics. *Eval. Res. Educ* 23 37–49. 10.1080/09500791003605102

[B69] Sorensen T. J. McKim A. J. (2014). Perceived work-life balance ability, job satisfaction, and professional commitment among agriculture teachers. *J. Agric. Educ.* 55 116–132. 10.5032/jae.2014.04116

[B70] Spreitzer G. M. (1995). Psychological empowerment in the workplace: dimensions, measurement, and validation. *Acad. Manag. J.* 38 1442–1465. 10.1111/jonm.12045 23815636

[B71] Srivastava S. Angelo K. M. (2009). “Optimism, effects on relationships,” in *Encyclopedia Of Human Relationships*, eds Reis H. T. Sprecher S. K. (Thousand Oaks, CA: Sage).

[B72] Thien L. M. Razak N. A. Ramayah T. (2014). Validating teacher commitment scale using a Malaysian sample. *Sage Open* 4:2158244014536744. 10.1177/2158244014536744

[B73] Tsang K. K. Wang G. Bai H. (2022). Enabling school bureaucracy, psychological empowerment, and teacher burnout: a mediation analysis. *Sustainability* 14 2047. 10.3390/su14042047

[B74] Tusaie K. R. Patterson K. (2006). Relationships among trait, situational, and comparative optimism: clarifying concepts for a theoretically consistent and evidence-based intervention to maximize resilience. *Arch. Psychiatr. Nurs.* 20 144–150. 10.1016/j.apnu.2005.10.004 16716858

[B75] Valeh M. Shokri O. Asadzadeh H. (2021). Effect of Teacher Psychological Empowerment Program on Teacher-Student Interaction. Emotions and Teachers Academic Optimism. *J. Instr. Eval.* 14 113–145.

[B76] Van Dop N. Depauw J. Driessens K. (2016). Measuring empowerment: development and validation of the service user psychological empowerment scale. *J. Soc. Serv. Res.* 42 651–664. 10.1080/01488376.2016.1216915

[B77] Wang S. Zhao Y. Cheng B. Wang X. Yang X. Chen T. (2018). The optimistic brain: trait optimism mediates the influence of resting-state brain activity and connectivity on anxiety in late adolescence. *Hum. Brain Mapp.* 39 3943–3955. 10.1002/hbm.24222 29923264 PMC6866307

[B78] Wang Y. L. Derakhshan A. (2021). [Review of the book Investigating dynamic relationships among individual difference variables in learning English as a foreign language in a virtual world, by M. Kruk]. *System* 100:102531. 10.1016/j.system.2021.102531

[B79] Xie F. Derakhshan A. (2021). A conceptual review of positive teacherinterpersonal communication behaviors in the instructional context. *Front. Psychol.* 12:708490. 10.3389/fpsyg.2021.708490 34335424 PMC8319622

[B80] Zimmerman M. A. (1990). Taking aim on empowerment research: on the distinction between individual and psychological conceptions. *Am. J. Community Psychol.* 18 169–177. 10.1007/BF00922695

[B81] Zimmerman M. A. (1995). Psychological empowerment: issues and illustrations. *J. Community Psychol.* 23 581–599. 10.1007/BF02506983 8851341

[B82] Zimmerman M. A. (2000). “Empowerment theory: psychological, organizational and community levels of analysis,” in *Handbook Of Community Psychology*, eds Rappaport J. Seidman E. (New York: Plenum Press), 43–63.

[B83] Zimmerman M. A. Israel B. A. Schulz A. Checkoway B. (1992). Further explorations in empowerment theory: an empirical analysis of psychological empowerment. *J. Community Psychol.* 20 707–727. 10.1007/BF01312604

